# Microvesicles: the functional mediators in sorafenib resistance

**DOI:** 10.20517/cdr.2021.137

**Published:** 2022-06-23

**Authors:** Cong He, Doulathunnisa Jaffar Ali, Bo Sun, Bei-Cheng Sun, Zhong-Dang Xiao

**Affiliations:** ^1^State Key Laboratory of Bioelectronics, School of Biological Science and Medical Engineering, Southeast University, Nanjing 210096, Jiangsu, China.; ^2^Department of Hepatobiliary Surgery, The Affiliated Drum Tower Hospital of Nanjing University Medical School, Nanjing 210008, Jiangsu, China.

**Keywords:** Microvesicles (MVs), sorafenib resistance, tumor microenvironment, cancer therapy

## Abstract

Overcoming drug resistance in cancer therapies remains challenging, and the tumor microenvironment plays an important part in it. Microvesicles (MVs) are functional natural carriers of cellular information, participate in intercellular communication, and dynamically regulate the tumor microenvironment. They contribute to drug resistance by transferring functional molecules between cells. Conversely, due to their specific cell or tissue targeting ability, MVs are considered as carriers for therapeutic molecules to reverse drug resistance. Thus, in this mini-review, we aim to highlight the crucial role of MVs in cell-to-cell communication and therefore their diverse impact mainly on liver cancer progression and treatment. In addition, we summarize the possible mechanisms for sorafenib resistance (one of the main hurdles in hepatocellular carcinoma treatments) and recent advances in using MVs to reverse sorafenib resistance in liver cancer therapies. Identifying the functional role of MVs in cancer therapy might provide a new aspect for developing precise novel therapeutics in the future.

## INTRODUCTION

Liver cancer (LC), including hepatocellular carcinoma (HCC), hepatoblastoma, and cholangiocarcinoma, remains one of the malignant cancers worldwide with high mortality^[1]^. Surgery, radiotherapy and chemotherapy are considered as the main treatments for LCs at present, but their therapeutic effects are limited in advanced stages^[2]^. Sorafenib, an oral drug approved by the FDA for the treatment of advanced HCC, has shown excellent therapeutic effects by inhibiting tumor growth and angiogenesis *in vitro*^[3,4]^. However, reported resistance limits its therapeutic effects^[5-9]^. Microvesicles (MVs), functional mediators in cell-to-cell communication by transferring bioactive cargoes^[10]^, play an important role in tumor progression and metastasis^[11-14]^. Interestingly, recent studies reported that HCC-derived MVs promoted sorafenib resistance in recipient liver cancer cells by transporting cancerous cargo compared to normal liver cell-derived MVs^[15]^. Thus, the role of these tiny functional vesicles needs to be cautiously studied for the in-depth understanding and successful treatment of HCC over sorafenib resistance.

Considering the necessity of overcoming sorafenib resistance while developing a successful treatment process for the challenging HCC, in the present review, we summarize the mechanism of action of sorafenib and sorafenib resistance. In addition, recent advances of MVs in intercellular communication and the intriguing contribution of MVs in cancer treatment are discussed. Especially, the review closely considers the possibilities for utilizing MVs as a potential therapeutic tool to alleviate sorafenib resistance in future cancer treatments. 

## SORAFENIB DRUG AND RESISTANCE

### Mechanism of action of sorafenib

**Figure 1:**
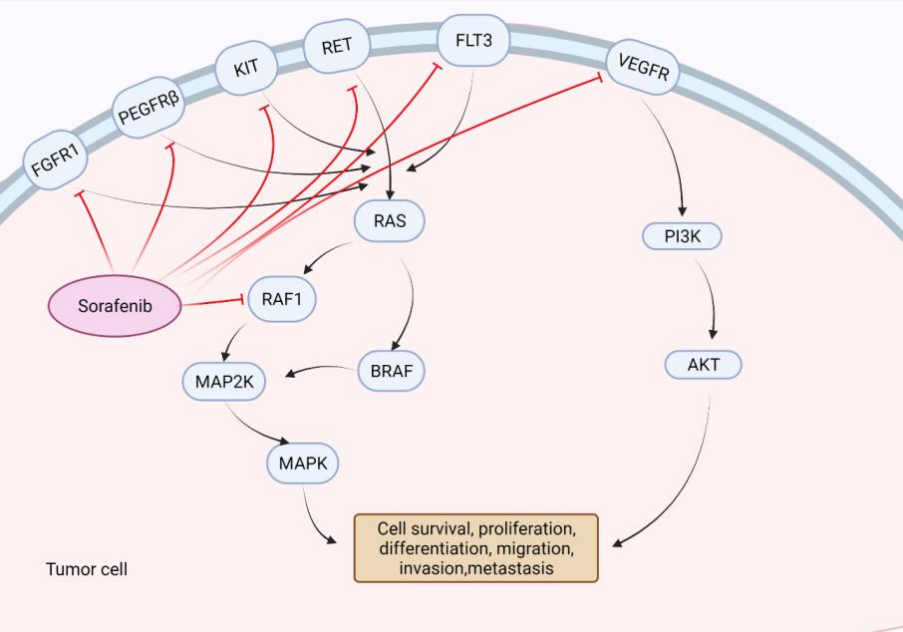
Mechanism of actions of sorafenib. Sorafenib inhibits tyrosine kinase receptor (VEGFR, PDGFRβ, Kit and RET) signaling and suppresses the activation of Raf, thus could suppress tumor progression by inhibiting angiogenesis and cell proliferation. Created with BioRender.com. VEGFR: Vascular endothelial growth factor receptor.

The revelation of the crucial involvement of Raf1 and vascular endothelial growth factor (VEGF) mediated signaling pathways in the molecular pathogenesis of liver cancer provided an interesting theoretic basis for applying sorafenib drugs to liver cancer treatment^[16,17]^. As a multikinase inhibitor, sorafenib strongly inhibits the tyrosine kinase Raf. Meanwhile, it has been shown to inhibit vascular endothelial growth factor receptor and platelet-derived growth factor receptor, which in turn inhibits the activation of other downstream multikinase that are normally essential for cell growth, angiogenesis, proliferation and metastasis of HCC cells [Figure 1]. Liu *et al.*^[18]^ recorded that sorafenib inhibited the proliferation of HCC cells and reduced angiogenesis signal transduction in HCC tumor xenograft, promoting tumor cell apoptosis as well. In addition, the same therapeutic advances have also been revealed in clinical studies^[19]^. Nevertheless, several studies stated that the therapeutic effects of sorafenib varied among patients, some of whom experienced severe side effects^[20-23]^. 

### Mechanism of sorafenib resistance

Drug resistance limits the therapeutic effects of HCC treatments. Roughly 30% of patients were reported to respond to sorafenib well at the beginning, while the subsequently acquired resistance to sorafenib usually happens within six months^[24]^, which is far from satisfactory. As an obvious contributor that hinders the effectiveness of cancer treatment, sorafenib resistance and the possible molecular mechanisms involved in sorafenib resistance become prominently important to be discussed here.

Due to the heterogenicity of liver cancer, some patients are resistant to sorafenib primarily, while others obtain sorafenib resistance during treatment, which further limits the application of the drug and leaves the treatment process questionable. Thus, the acquired resistance of sorafenib attracted the attention of a wide range of researchers^[8,25]^. To date, studies on the potential mechanism of sorafenib resistance have mainly focused on the activation of drug targets and downstream signaling, regulation of cell proliferation and apoptosis signaling^[5-9]^. In addition, stemness and mesenchymal states of sorafenib-resistant HCC cells provided a new aspect of this challenging problem^[26]^. Altogether, the mechanism of sorafenib resistance and its influence on the treatment process remain complicated and require more research for a better understanding.

The epidermal growth factor receptor (EGFR) has been found to be overexpressed or hyperactivated in the cancer cells of most liver cancer patients as well as be the reason for continuous activation of its downstream signaling of the Ras/Raf/MEK/ERK pathway. This contributes to the abnormal proliferation of cancer cells and therefore might promote sorafenib resistance^[27]^. For instance, an attenuated level of phosphorylated ERK was reported to be associated with sorafenib resistance in HCC^[28]^. In addition, hyperactivated EGFR/HER3 and its overexpressed ligands were reported to suppress the curative effect of sorafenib by interfering with the phosphorylation of EGFR/HER3, by which the enhanced anti-proliferative and pro-apoptotic abilities of sorafenib could be achieved during the treatment^[29]^. 

A body of evidence reveals that the PI3K/Akt pathway plays an important role in sorafenib resistance^[5,30]^. For instance, Chen *et al.*^[5]^ found that exposure of Huh7 liver cancer cells to a high concentration of sorafenib could result in sorafenib resistance and an accelerated expression of Akt in the treated cells. Furthermore, the PI3K/Akt pathway has been identified to have a close relationship with cell apoptosis. In the pathway, the combination of pro-survival factor and tyrosine kinase receptor activates the kinase PI3K, which triggers the downstream cascade to endorse phosphorylation of Akt and thus contributes to the suppression of cell apoptosis. In turn, inhibition of Akt could make the tumor cell more responsive to sorafenib treatment^[31]^. Hence, silencing of PI3K/Akt signaling with Akt inhibitor alone or with other combination therapy has gained attention for reversing sorafenib resistance for better HCC treatment^[32,33]^. Furthermore, Src homology 2 domain-containing protein tyrosine phosphatase 1 (SHP-1) has been reported to be activated by sorafenib, which in turn could negatively regulate pSTAT3 and suppress transduction of JAK/STAT signaling. Dysfunctional JAK/STAT has been observed in sorafenib-resistant liver cancer cells, including induced expression of pSTAT3 and its downstream anti-apoptotic protein Mcl-1 and reduced expression of SHP-1/pSHP-1^[34]^.

Moreover, cancer stem cells (CSCs), which represent a subpopulation of cancer cells with a self-renewal nature, are considered to participate in tumorigenesis, drug resistance, tumor metastasis and recurrence, and they are innovative targets for cancer therapy^[35-37]^. Label-retaining cancer cells (LRCCs) can be used to label CSCs. Xin *et al.*^[38]^ used this methodology to observe sorafenib treated HCC cells and found that LRCCs were highly enriched in the remaining HCC cells that escaped sorafenib treatment, which strongly evidenced the resistance to sorafenib-induced cytotoxicity and apoptosis. In addition, specific ATP binding box (ABC) transporters have been reported to be highly expressed on CSCs, which control the outflow of chemical agents to protect cells from toxic compound accumulation and damage, and hence could reduce the sensitivity of cells towards drug treatment^[39,40]^. ABCB1 has been attested to have a close relationship with multidrug resistance; thereby, knocking out ABCB1 in drug-resistant cancer cell lines made those cells more responsive to chemotherapies^[41]^. It was also revealed that CSCs isolated from HCC cell lines showed resistance to sorafenib both* in vitro* and* in vivo* with abnormal IL-6/STAT3 signaling^[6]^. Through VEGF, liver cancer stem cells could promote tumor angiogenesis to sustain their stemness as well as drug resistance features^[42]^. In addition, Wnt/β-catenin signaling, one of the classic pathways involved in stemness regulation^[43,44]^, was proven to be hyperactivated in HCC cells, resulting in β-catenin accumulation in cytoplasm and nucleus, which finally led to enhanced self-renewal ability of CSCs^[45,46]^. Inhibition of Wnt signaling has been shown to be beneficial to CSC clearance and tumor development^[46,47]^.

Dual biotransformation routes including oxidation and glucuronidation were witnessed in the sorafenib metabolism^[48-50]^. After hepatocellular uptake, sorafenib was N-oxidized by CYP3A4, one of the drug-metabolizing enzymes, to the pharmacologically active sorafenib-N-oxide metabolites^[51,52]^. However, CYP3A4 was identified as poorly expressed in liver cancer^[53]^. Well-studied oncomiRs (e.g., miR 21, miR-142 and miR-27b) overexpressed in HCC, which could be transferred by tumor-derived microvesicles (TMVs), were proved to be negatively associated with CYP3A4 mRNA in human liver^[54]^, which might downregulate the expression of this main enzyme and thus could inhibit the active biotransformation of sorafenib drug. Apart from oxidation, sorafenib underwent glucuronidation, mainly mediated by UGT1A1 and UGT1A9, to inactive glucuronide metabolites^[48,55]^. A recent study revealed that sorafenib inhibits the above-mentioned UGT enzyme^[56]^, which might be the blockage for sorafenib secretion to bile and later systemic circulation and clearance. Taken together, the insufficient oxidation and complex interaction between sorafenib and UGT enzymes need further investigation for a deep understanding of their role in resistance.

Anti-angiogenesis is one of the therapeutic effects of sorafenib, while the tumor vessel depletion along with pericyte could induce hypoxia and allow the maintenance and enhancement of CSCs in HCC^[57,58]^. Apart from specific niches, different stroma cells in the microenvironment render sorafenib sensitivity^[59]^, where MVs play a significant role.

## MVS: THE TINY MEDIATORS IN INTERCELLULAR COMMUNICATION

MVs, a subpopulation of extracellular vesicles (EVs), act as functional mediators by transferring bioactive molecules among various types of cells and thus have been considered as potential candidates in intercellular communication^[10]^. Through unconventional secretion mechanisms, eukaryotic cells were reported to release membrane-enclosed vesicles both *in vivo* and *in vitro*^[60]^. In general, MVs can be collected from cell culture media and blood from animals and patients via ultracentrifuge method, filtration, or commercial kits^[61]^. MVs are also identified as shedding microvesicles or microparticles, budding directly from the plasma membrane and are 100 nm-1000 nm in diameter. They participate in cell-to-cell communication by carrying the information from parental cells to others and orchestrate complicated physiological and pathological processes^[14,62]^. At the molecular level, symmetrical perturbation of membrane lipids leads to the surface expression of phospholipid serine acid pairs, which translocate from the inner lobule to the outer surface lobule of the membrane bilayer through special biological enzymes (floppases, flippases and scramblases), contributing to MVs formation^[63]^. However, the detailed mechanism of MVs formation and shedding remains to be further studied. 

Evidence reveals that most MVs tend to be decomposed after shedding to release their cargo^[64]^. Especially, various cytoplasmic proteins, excluded from the classic signal-peptide secretion pathways, are discharged out regularly through MV decomposition^[65]^. For instance, fibroblast growth factor 2 (FGF2) was proved to be released in response to the breakdown of MVs derived from neurons, HCC and endothelial cells^[66]^. Similarly, MVs secreted by dendritic cells, macrophages and microglias have been demonstrated to release the pro-inflammatory cytokine interleukin 1B (IL-1B); IL-1B has also been found to aggregate in MVs along with proteases such as caspase 1^[67]^. A study on tumor-stromal interactions noted that when MVs were co-released with extracellular matrix metalloproteinase inducer (EMMPRIN/CD147), they prompted lung cancer cells to obtain increased mobility and invasion ability by promoting metalloproteinase to capture and digest extracellular matrix (ECM)^[68]^. In addition, Kornek *et al.*^[69]^ demonstrated that EMMPRIN released by circulating T cell-derived MVs accelerated hepatic stellate cells to transform into a fibrolytic phenotype, which promotes ECM degradation.

Conversely, MVs are able to stimulate the receptor cells to complete signal transduction through their surface ligands. It has been confirmed that MVs derived from platelet arouse the intra-hemopoietic signal cascade by ligands such as CD40L/PF-4 and thus cause the proliferation and survival of hematopoietic cells^[70]^. Simultaneously, the adhesion molecules could be transferred to hematopoietic cells via MVs to enhance their adhesion to fibrinogen or endothelium^[70]^. Proteomic and transcriptomic studies have shown that MVs are natural vectors for transferring bioactive molecules, including protein, mRNA and miRNA, between cells^[71]^, effectively facilitating intercellular communication^[72,73]^. In addition, MVs hold natural stability in the blood, low immunogenicity and special homing ability to specific organs or tissues^[74]^. Evidence shows that vesicle-associated integrins α6β4 and α6β1 are correlated with lung metastasis while αVβ5 with liver metastasis, which indicates the various origins of MVs encoded with different tags for the corresponding cells or organ targeting^[74]^. Moreover, it has also been demonstrated that MVs could possibly transport even complete organelles (e.g., mitochondria) to target cells^[75]^, which declares its potential to act as a promising novel drug carrier system.

**Figure 2:**
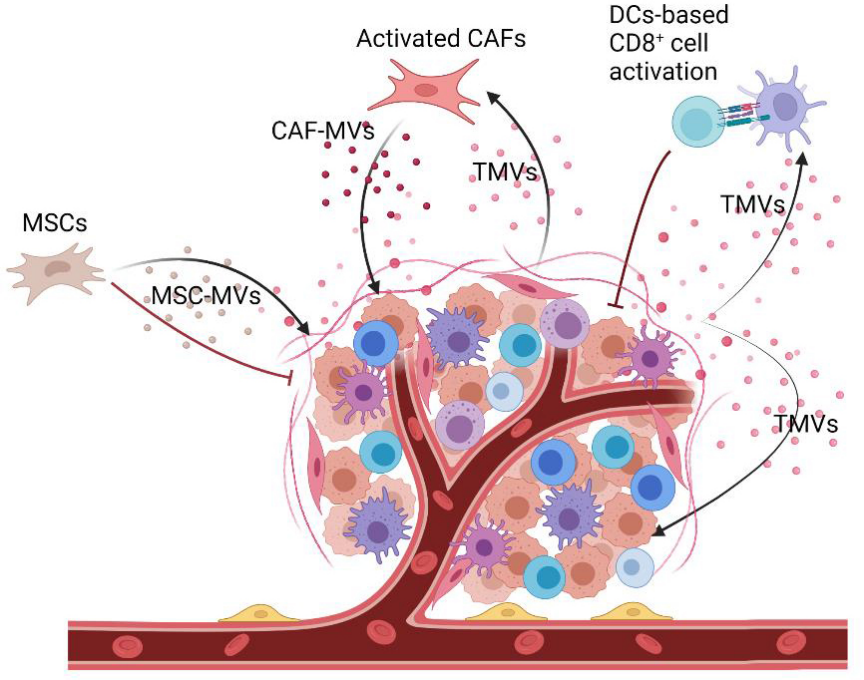
The bidirectional role of TMVs in tumor development. TMVs derived from tumor cells could promote tumor progression via transferring cancerous molecules^[13,62]^. Simultaneously, they act as a functional regulator to modulate DC cells based on CD8^+^ T cell activation, which provokes cytotoxic T cell infiltration and inhibits tumor development^[77]^. Created with BioRender.com. TMVs: Tumor-derived microvesicles.

Several studies revealed that TMVs transport cancerous molecules to recipient cells, which eventually contribute to tumor progression and metastasis^[11-14,76] ^[Figure 2]. Interestingly, the transport of anti-cancer drugs out of ovarian cancer cells through their secreted EVs supported the role of EVs in cancer progression and treatment. Conversely, the observation of more sorafenib resistance in HCC tumors that were treated with tumor cell-derived MVs added evidence to the noticeable link between MVs and sorafenib resistance^[15,78]^. 

## THE DYNAMIC IMPACT OF MVS ON SORAFENIB RESISTANCE

### MV-mediated sorafenib resistance

The physiological status of parental cells decides the composition of their secreted MVs, having direct or indirect effects on the uptake of MVs by recipient cells. Tumor cells that undergo hypoxic stress and obtain stemness or mesenchymal state for survival^[57,58]^ promote tumor progression and therapy resistance. By inducing hypoxic stress, the hypoxia-inducible factor-1 α could stimulate the release of MVs along with the modulation of its packed cargoes^[79]^. Similarly, Wang *et al.*^[80]^ reported that chemotherapeutic agents could enhance the secretion of ABCB-1-enriched EVs, which promote resistant phenotype transformation in recipient cells. Specifically, growth and pro-angiogenic factors transferred between CSCs and vascular niches via vesicles under hypoxia were witnessed^[81]^ to limit the therapeutic effect of sorafenib.

Cancer-associated fibroblasts (CAFs), one of the main stroma cells, advance the self-renewal feature of CSCs in HCC and thus could induce sorafenib resistance by secretion of hepatocyte growth factor (HGF)^[82,83]^. A reduction in cancer cell stemness was recorded by inhibiting the paracrine behavior of CAFs^[84]^. Conversely, TMVs have been shown to activate CAFs to improve the mobility of tumor cells, while the activated fibroblasts could secret MVs and in turn facilitate tumor progression^[12,85]^. Moreover, small GTPases and RHO-associated protein kinase, key factors in the biogenesis of MVs^[73]^, were found to be highly expressed in both CAFs and cancer cells^[86]^, which indicates the possible role of CAFs in the alteration of MVs secretion.

**Figure 3:**
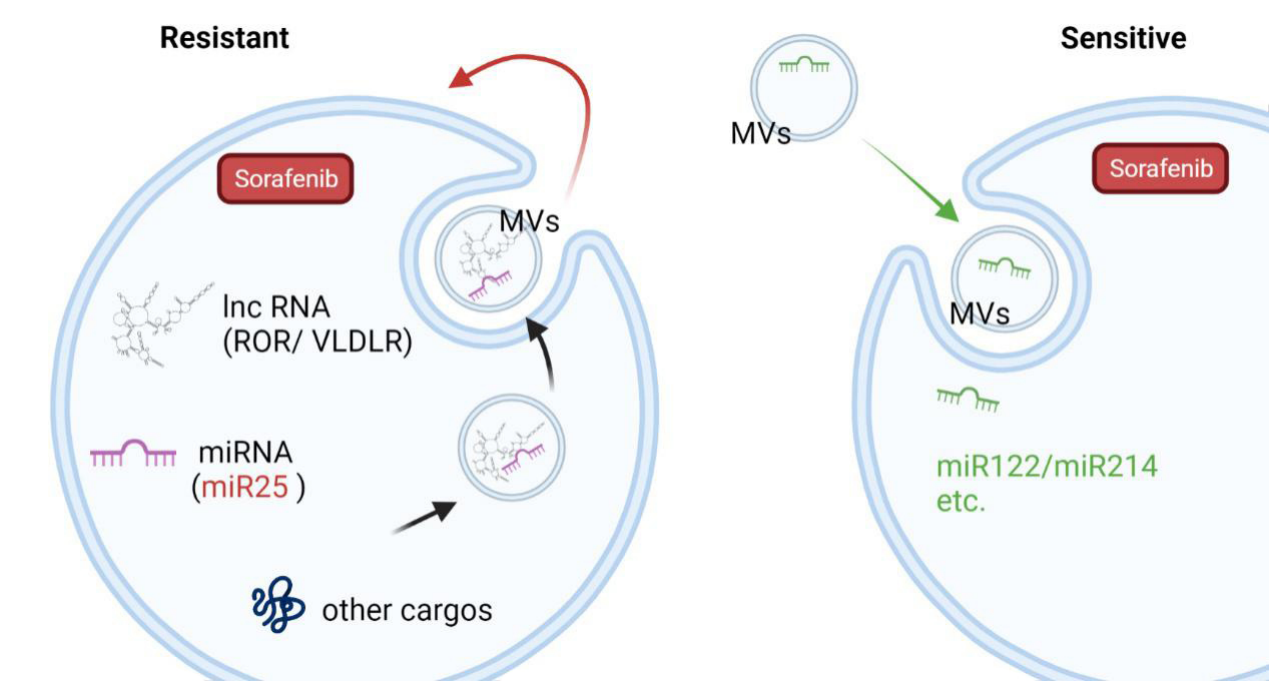
The dynamic role of MVs in sorafenib resistance modulation. MVs transfer biomolecules including miRNAs and lncRNAs to recipient cells and regulate their sensitivity to sorafenib. Created with BioRender.com. MVs: Microvesicles.

As the messengers and mediators between cells, MVs could regulate the sorafenib sensitivity in the recipient cells via their cargoes [Table 1]. For example, miRNAs involved in the regulation of multiple mRNA targets in recipient cells were found to be enriched in TMVs^[94-97]^. In recent years, our group also found that HCC cell-derived MVs could increase sorafenib drug resistance by inducing FOXM1 expression via miR-25 transferred to the recipient liver cancer cells from the parent cells both *in vitro* and *in vivo*^[15]^. Similarly, long non-coding RNA (lnc-ROR and lnc-VLDLR)-enriched HCC cells-secreted vesicles (especially after sorafenib exposure) were proved to reduce apoptosis induced by chemotherapy^[88,89]^. However, MVs released from modified adipose tissue-derived MSCs have been shown to carry miR-199a/miR-122 and have the ability to improve chemosensitivity in HCC^[90,91]^. Elevated miR-214 in human cerebral endothelial cell-released vesicles was noted to enhance the anti-tumor efficacy of sorafenib^[92]^ [Figure 3]. However, complete genomic and proteomic analyses of preclinical MVs cargoes need to be performed in the future.

**Table 1:** Key molecules transferred by MVs modulating sorafenib resistance

**Key molecules**	**MVs sources**	**Function**	**Ref.**
miR-25	HCC cells	Increase sorafenib resistance	[15]
miR-494-3p	GOLPH3 overexpressed HCC cells	Promote the angiogenesis ability of HUVECs and induce sorafenib resistance in HCC cells	[87]
lnc-ROR	HCC cells	Reduce chemotherapy-induced cell death	[88]
lnc-VLDLR	HCC cells	Reduce chemotherapy-induced cell death	[89]
miR-199a	Adipose tissue derived MSCs	Increase chemosensitivity in HCC	[90]
miR-122	Adipose tissue derived MSCs	Increase chemosensitivity in HCC	[91]
miR-214	Human cerebral endothelial cells	Sensitize HCC cells to sorafenib treatment	[92]
siGRP78	Modified bone-marrow-derived mesenchymal stem cells	Suppress sorafenib resistance	[93]

MVs: Microvesicles; HCC: hepatocellular carcinoma.

### Targeting MVs to reverse sorafenib resistance

Recently, the promising outcome of targeted gene therapy has motivated more researchers to meet sorafenib resistance in HCC. A lower expression of miR-34 has been found to have a direct relationship with patient survival, while the restoration of the miR-34a expression level in HCC cells has been noted to significantly downregulate the expression of BCL2 and enhance the cells’ sensitivity towards sorafenib-induced apoptosis and toxicity^[7]^. Dai *et al.*^[98]^ found that BikDD gene therapy combined with low-dose sorafenib could enhance the anti-tumor efficacy of sorafenib and improve the survival rate of tumor-bearing mice. Furthermore, the simultaneous release of sorafenib and USP22 shRNA (shUSP22) by galactose-modified lipopolysaccharide revealed that the encapsulated sorafenib along with shUSP22 could obtain a synergistic anti-proliferative effect in HCC cells by inducing reactive oxygen cascade to promote the release of shUSP22 and inhibit the expression of USP22 in HCC cells, which promoted the accumulation of sorafenib by downregulating the expression level of multidrug resistance-related proteins^[99]^. 

Several emerging studies reported utilizing modified EVs to overcome chemoresistance during cancer treatments. As a naturally secreted nanoparticle, MVs exhibited excellent specific tissue homing ability and acted as good functional carriers for various therapeutic molecules^[15,74,100]^. To achieve bioactive and continuous drug containing MVs, the pre-loading strategy has been applied to modify donor cells. This strategy includes incorporating cargo into donor cells, so the donor cells could encapsulate the cargo during secretion production. Both biologically produced molecules (proteins and nucleic acids) and synthetic chemicals can be encapsulated into secreted vesicles^[2]^. By combined regulation of Akt/mTOR/PTEN, EVs secreted from human liver stem cells were proved to upgrade CSCs’ sensitivity to sorafenib^[101]^. Lou *et al.*^[91]^ stated that treating HCCs with exosomes derived from miR-122 overexpressed AMSCs rendered HCCs more responsive to sorafenib. In the same way, miR-744-enriched exosomes have been demonstrated to be a potential tool for reducing sorafenib resistance^[102]^. Apart from utilizing the intrinsic homing ability, controllable targeting methods, including genetic engineering (pre-targeting)^[103]^ and conjugation of ligands (post-targeting)^[104]^, have also been developed for further clinical research. Engineering the donor cells by inserting sequences that encode desired targeting protein makes the affibody express on the surface of MVs^[105]^. In this way, our group also recently recorded that CRISPR system-carried engineered MVs derived from HEK293 cells could precisely disrupt IQGAP1, which is involved in PI3K/Akt signaling, and achieved an enhanced synergistic anti-cancer effect when combined with sorafenib treatment^[106]^.

## CONCLUSION

Sorafenib resistance remains challenging in HCC treatment, while targeting the suspicious genes involved in the resistance mechanism to shut off the pathological cycle provides a new chance for advancing the cancer treatment as well as the existing anti-cancer drug^[107]^. For instance, by delivering sorafenib and CRISPR system via nanoparticles, Zhang *et al.*^[108]^ achieved effective modification of EGFR, following synergistic inhibition of angiogenesis and tumor cell proliferation. Even though emerging synthetic nanomaterials have shown great abilities as therapeutic vectors, their long-term safety remains uncertain.

MVs, cell-derived natural carriers, are advanced promising gene therapy vehicles that could specifically deliver therapeutic bioactive molecules^[109]^. Platelet-derived MVs have been reported to hold good biocompatibility to target leukemia cells naturally and thus have been utilized as targeted delivery vehicles for multiple drugs in leukemia treatment^[110]^. Macrophage-derived MVs have been noted to have the ability to transport cargoes specifically to hematopoietic stem and progenitor cells (HSPCs) both *in vivo* and *in vitro*. In addition, Kao *et al.*^[100]^ showed that plasmids and small RNAs (miRNA and siRNA) that were encapsulated by macrophage-derived MVs exhibit successful modification of heat shock protein in the recipient HSPCs. In addition, MVs were used to transport engineered minicircle DNA by researchers to achieve good gene-mediated prodrug transformation and effectively promote tumor cell death in breast cancer cells as well as mouse models^[111]^. However, the unexpected combination, modification, and dissociation of the above-mentioned therapeutic nucleic acids are the main concerns in gene manipulation techniques, in which the off-target effect needs to be well controlled in future clinical trials.

Considering the challenges of sorafenib resistance and MVs as the functional regulator in cancer microenvironment, in this review, we discuss the potential role of MVs in sorafenib resistance. On the one hand, in cancer progression, MVs transport bioactive molecules to participate in cell-to-cell communication, making the microenvironment favor tumor growth, which could facilitate the tumor cells to be resistant to sorafenib treatment. On the other hand, therapeutic molecules could be transferred specifically to tumor cells to alleviate the sorafenib resistance via MVs. Thus, a better understanding of these tiny players’ role in tumor microenvironment and cancer progression is necessary to appropriately use this double-edged sword for precise anti-cancer therapies in the future.

## DECLARATIONS

### Copyright

© The Author(s) 2022.
